# Endosperm and Seed Transcriptomes Reveal Possible Roles for Small RNA Pathways in Wild Tomato Hybrid Seed Failure

**DOI:** 10.1093/gbe/evab107

**Published:** 2021-05-19

**Authors:** Ana Marcela Florez-Rueda, Flurin Fiscalini, Morgane Roth, Ueli Grossniklaus, Thomas Städler

**Affiliations:** 1 Department of Plant and Microbial Biology & Zurich–Basel Plant Science Center, University of Zurich, 8008 Zurich, Switzerland; 2 Institute of Integrative Biology & Zurich–Basel Plant Science Center, ETH Zurich, 8092 Zurich, Switzerland

**Keywords:** postzygotic isolation, small RNAs, seed development, epigenetic, transcriptomics, *Solanum*

## Abstract

Crosses between the wild tomato species *Solanum peruvianum* and *Solanum chilense* result in hybrid seed failure (HSF), characterized by endosperm misdevelopment and embryo arrest. We previously showed that genomic imprinting, the parent-of-origin–dependent expression of alleles, is perturbed in the hybrid endosperm, with many of the normally paternally expressed genes losing their imprinted status. Here, we report transcriptome-based analyses of gene and small RNA (sRNA) expression levels. We identified 2,295 genes and 387 sRNA clusters as differentially expressed when comparing reciprocal hybrid seed to seeds and endosperms from the two within-species crosses. Our analyses uncovered a pattern of overdominance in endosperm gene expression in both hybrid cross directions, in marked contrast to the patterns of sRNA expression in whole seeds. Intriguingly, patterns of increased gene expression resemble the previously reported increased maternal expression proportions in hybrid endosperms. We identified physical clusters of sRNAs; differentially expressed sRNAs exhibit reduced transcript abundance in hybrid seeds of both cross directions. Moreover, sRNAs map to genes coding for key proteins involved in epigenetic regulation of gene expression, suggesting a regulatory feedback mechanism. We describe examples of genes that appear to be targets of sRNA-mediated gene silencing; in these cases, reduced sRNA abundance is concomitant with increased gene expression in hybrid seeds. Our analyses also show that *S. peruvianum* dominance impacts gene and sRNA expression in hybrid seeds. Overall, our study indicates roles for sRNA-mediated epigenetic regulation in HSF between closely related wild tomato species.

Significance

Hybrid seed failure (HSF) constitutes a widespread and potentially fast-evolving reproductive barrier between closely related species of flowering plants. There is mounting evidence that epigenetic asymmetries between the parents may lead to expression alterations in the developing endosperm that result in seed abortion. Here, we use small-RNA and gene expression patterns obtained from seeds derived from within- and between-species crosses with a species pair of wild tomatoes with near-complete HSF. Differential gene expression analyses reveal that hybrid seeds have lower small-RNA transcript abundance than “normal” seeds, while many associated genes show higher hybrid expression. Our data implicate small-RNA pathways as playing a functional role in patterns of hybrid gene expression and can be connected to phenomena recently uncovered in the model plant *Arabidopsis*.

## Introduction

The establishment of reproductive barriers between diverging lineages is a basic component of the speciation process and thus of major interest in evolutionary biology (Coyne and Orr 2004). In this study, we assess the molecular correlates of hybrid seed failure (HSF), a form of postzygotic barrier acting early in the seed development of many flowering plants (Städler et al. 2021). In the angiosperm seed, embryo and endosperm are the products of two independent fertilization events. The endosperm is usually a triploid tissue with two maternal to one paternal genome ratio (2m:1p) nourishing the growing embryo; failure of proper endosperm development often leads to embryo arrest and is considered the main cause of HSF (Rebernig et al. 2015; Garner et al. 2016; Oneal et al. 2016). HSF has frequently been observed upon hybridization of closely related homoploid plant species as well as between lineages differing in ploidy (Beamish 1955; Johnston et al. 1980; Scott et al. 1998; Rebernig et al. 2015; Baek et al. 2016; Sandstedt et al. 2021; Städler et al. 2021).

From an evolutionary perspective, the developing seed can be viewed as an arena in which the “interests” of two parental genomes “collide.” Any differences in parental optima for resource allocation to the progeny (representing a parental conflict) are expected to manifest themselves in the endosperm (Haig and Westoby 1991; Haig 2013). The ratio of “effective” parental genomic contributions in the endosperm appears to largely determine the success or failure of particular crosses, an interpretation bolstered by the frequent observation that postzygotic barriers can be weakened by manipulating the ploidy of one of the parents (Johnston et al. 1980; Josefsson et al. 2006; Lafon-Placette and Köhler 2016). Transgressive and complementary hybrid seed phenotypes are common and thought to reveal different levels of parental conflict between lineages (Lu et al. 2012; Haig 2013; Rebernig et al. 2015; Florez-Rueda, Paris, et al. 2016; Lafon-Placette et al. 2018; Städler et al. 2021). These observations have led to the hypothesis that parent-of-origin–dependent allelic expression (i.e., genomic imprinting) might be causally involved in HSF. Genomic imprinting is an epigenetic phenomenon causing the preferential expression of alleles depending on their parental origin. In flowering plants, while occurring also in the embryo (Jahnke and Scholten 2009; Raissig et al. 2013), genomic imprinting is prevalent in the endosperm and critical for proper seed development (Grossniklaus et al. 2001; Gehring and Satyaki 2017; Batista and Köhler 2020).

Although perturbed genomic imprinting has been shown to be a molecular correlate of HSF (Josefsson et al. 2006; Walia et al. 2009; Jullien and Berger 2010; Burkart-Waco et al. 2015; Wolff et al. 2015; Florez-Rueda, Paris, et al. 2016), successful seed development results from the precise orchestration of additional genomic and developmental processes. Other molecular processes during seed formation, such as the derepression of transposable elements (TEs; Fultz et al. 2015; Martínez and Köhler 2017) and gene regulation mediated by small RNAs (sRNAs; Lu et al. 2012; Ng et al. 2012), likely act in the endosperm to determine the success or failure of particular cross combinations. Of particular interest are sRNAs; these RNA forms are involved in plant development, reproduction, and genome reprogramming (Haig 2013; Benkovics and Timmermans 2014; Borges and Martienssen 2015; Martínez and Köhler 2017; Satyaki and Gehring 2019; Paro et al. 2021).

For instance, microRNAs (miRNAs) are post-transcriptional regulators of gene expression, and various other types of sRNAs are involved in post-transcriptional gene silencing (PTGS) via transcript cleavage or translational repression as well as in transcriptional gene silencing (TGS), the latter mostly via RNA-directed DNA methylation (RdDM; Matzke and Mosher 2014; Pikaard and Mittelsten Scheid 2014; Borges and Martienssen 2015; Cuerda-Gil and Slotkin 2016; D’Ario et al. 2017). Several recent studies point to a pivotal role for sRNA-mediated gene silencing in regulating proper seed development and/or hybrid fitness (Groszmann et al. 2011; Lu et al. 2012; Rodrigues et al. 2013; Vu et al. 2013; Martínez et al. 2016, 2018; Borges et al. 2018; Satyaki and Gehring 2019). Although current knowledge regarding sRNA biogenesis and regulatory mechanisms stems mainly from work in the model species *Arabidopsis thaliana* and other Brassicaceae (Grover et al. 2020; Wang et al. 2020), it is expected that the underlying concepts apply to most angiosperms. However, some deviations from the canonical mechanisms may occur in more distantly related taxa, such as our study system *Solanum*.

In this study, we quantified the expression patterns of sRNAs in reciprocal crosses between two wild tomato species that show near-complete HSF, an important postzygotic barrier to interbreeding among several species of wild tomatoes (*Solanum* section *Lycopersicon*). Classical studies found high proportions of HSF in reciprocal crosses between the closely related *Solanum peruvianum* (P) and *Solanum chilense* (C) (Rick and Lamm 1955). Following this pioneering work, we have quantified various degrees of seed inviability in reciprocal hybrid crosses involving several species of wild tomatoes. Moreover, we observed differences in the cellular architecture and histology of failing endosperms, as well as strong differences in seed size depending on the direction of hybrid crosses (Roth, Florez-Rueda, Griesser, et al. 2018). Similar HSF-associated phenotypes have been described in different *Solanum* species and other angiosperm taxa, including interploid and homoploid hybrid crosses in model species and important crops (Cooper and Brink 1945; Beamish 1955; Scott et al. 1998; Dilkes et al. 2008; Ishikawa et al. 2011; Burkart-Waco et al. 2013; Rebernig et al. 2015; Roth et al. 2019; Coughlan et al. 2020; Städler et al. 2021).

We previously studied the molecular correlates of HSF in reciprocal *S. peruvianum* × *S. chilense* crosses and found that genomic imprinting in the endosperm is systematically perturbed (Florez-Rueda, Paris, et al. 2016), but we did not assess changes in overall expression levels. This intriguing pattern motivated us to investigate the likely epigenetic basis of strong HSF as observed in *S. peruvianum* × *S. chilense* crosses, with a focus on the possible roles of sRNAs. In the present study, we integrate gene and sRNA expression estimates and assess their expression profiles in both normally developing and failing hybrid endosperm and seeds, respectively. We examine the targets of the sRNAs and provide examples of representative genes exhibiting changes in gene expression concomitant with sRNA expression variation. By comparing the expression patterns of reciprocal hybrids and their parents, we further test how expression inheritance patterns are shaped by different “effective ploidies” of the parental lineages.

## Results

### Mapping and Gene Identification

We performed sRNA sequencing from whole seeds obtained from intra- and reciprocal interspecific crosses. Three replicate sets of “normal” and “hybrid” sRNA transcriptomes were produced for each of the two main parental plants, the same individuals we used in our previous study (supplementary fig. S1, Supplementary Material online; Florez-Rueda, Paris, et al. 2016). After sequencing, we obtained a mean of 9.6 million reads per library, of which a mean of 45.6% were kept after quality filtering and mapping (supplementary table S1, Supplementary Material online). Based on ShortStack’s (Axtell 2013; Johnson et al. 2016) default criteria for the identification of sRNA clusters, we report all identified 61,697 sRNA clusters with complete annotation and expression estimates (supplementary table S2, Supplementary Material online). Of these, we kept 31,189 that fell within 2.5-kb flanking regions of protein-coding genes. Not surprisingly, the majority of sRNA clusters comprise 24-nt sRNAs (namely, 27,202 clusters), whereas only 1,594 correspond to 21–22-nt sRNA clusters. To integrate sRNA and gene expression information, we remapped our previously produced endosperm transcriptomes obtained after Laser-Assisted Microdissection (LAM; Florez-Rueda, Paris, et al. 2016) to the *Solanum lycopersicum* reference genome. A mean of 21 million reads per library mapped uniquely to the reference genome and was used in subsequent analyses, making the mean proportion of retained reads 84% of the initially obtained raw data (supplementary table S1, Supplementary Material online). We thus detected 33,805 transcripts across all endosperm transcriptomes.

### Differential Expression in Hybrid Endosperms of Wild Tomatoes

**Fig. 1.**
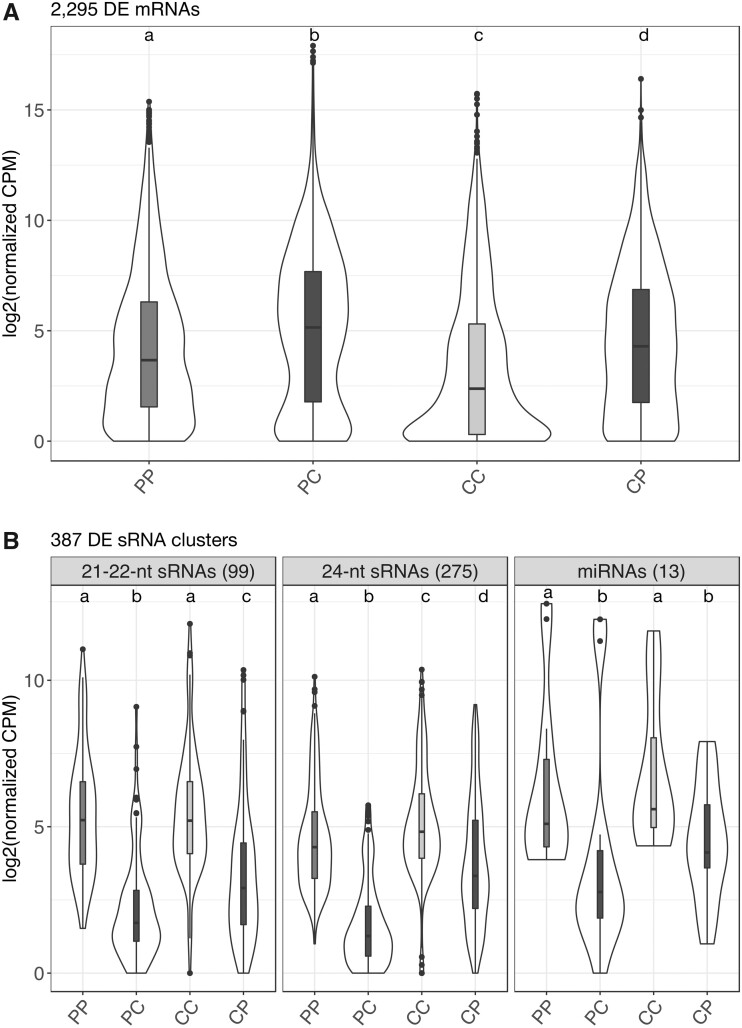
Expression distributions of 2,295 differentially expressed genes in the endosperm (A), and 387 sRNA clusters in whole seeds (B) differentially expressed between within-species and between-species hybrid crosses (contrast [PP, CC] vs [PC, CP]). Plants LA1616A (P) and LA4329B (C) served as maternal plants in both cross types. Letters on top of violin plots represent significant differences between expression distributions (Wilcoxon rank-sum test, *P *<* *0.01). CPM, counts per million.

We identified common trends of differential expression between normal and hybrid endosperms, with LA1616A (P) and LA4329B (C) serving as maternal parents in both cross types (contrast [PP, CC] vs [PC, CP]). Genes that are consistently differentially expressed (DE) in the hybrid endosperms of both species tend to have higher levels of expression when compared to ‘normal’ (intraspecific) endosperms in each species (figure 1*A*, Wilcoxon rank-sum test <2e−16 in all normal vs hybrid comparisons). Of the 33,805 transcripts for which we obtained expression values, 2,295 were found as DE in hybrid endosperms; transcripts identified as DE are reported in supplementary table S3, Supplementary Material online. Of these, 1,515 were found overexpressed and 780 underexpressed in the hybrid compared to normal endosperms from the same maternal plants.

To test the possible roles of sRNAs in mediating the increases in gene expression (fig. 1*A*) and maternal allelic proportions (Florez-Rueda, Paris, et al. 2016), we investigated patterns of sRNA expression. The pattern of whole-seed sRNA differential expression is in stark contrast to the increase in gene expression we found among DE genes in hybrid endosperms. From the 31,189 total sRNA clusters identified across all sRNA libraries and present within 2.5-kb gene boundaries, only 387 clusters were DE. These correspond to miRNAs (*n *=* *13), 24-nt sRNAs (*n *=* *275), and 21–22-nt sRNAs (*n *=* *99) (fig. 1*B*; supplementary table S4, Supplementary Material online). Their altered expression is consistent in reciprocal hybrid crosses, with sRNAs being underexpressed in both PC and CP hybrid seeds (Wilcoxon rank-sum test <0.01 in all normal vs hybrid comparisons). Differences in sRNA expression are larger in seeds from *S. peruvianum* maternal plants (fig. 1*B*), thus mirroring the differences in seed phenotype and increases in maternal allelic proportions in hybrid endosperms, which both are more marked in hybrid seeds with *S. peruvianum* as the maternal parent (Florez-Rueda, Paris, et al. 2016; Roth, Florez-Rueda, Griesser, et al. 2018).

To shed light on the roles of a putative RdDM pathway in *Solanum*, we examined patterns of expression of the principal subunits of RNA polymerases Pol IV, Pol V, and Pol II in hybrid versus normal *Solanum* seeds (supplementary table S5, Supplementary Material online). We observed reduced hybrid expression of both genes encoding the subunits of Pol IV: RNA polymerase 4 largest subunit, *RPD1* (log fold-change [FC] = −1.82, false discovery rate [FDR]-corrected *P *=* *9.69E−49), and RNA polymerase 4 second largest subunit, *RPD2* (logFC = −0.56, FDR-corrected *P *=* *0.0148), as well as reduced expression of the gene encoding subunit H of Pol V (logFC= −2.09, FDR-corrected *P *=* *1.75E−55).

The general pattern of overexpression in hybrid endosperms holds particularly for genes coding for transcription factors (TFs; supplementary table S6, supplementary fig. S2*C*–*F*, Supplementary Material online). Genes encoding subunits of the mediator complex, a global regulator of Pol II, were found overexpressed in hybrid endosperms, with the term IPR013921, mediator complex significantly enriched. Overexpression is higher in the hybrid endosperm of *S. chilense* than of *S. peruvianum* maternal parents (supplementary fig. S2*D*, Supplementary Material online), with many of these genes belonging to the term GO: 0001104, RNA polymerase II transcription cofactor activity. Strikingly, we uncovered consistent overexpression of 29 genes containing a MADS-box (IPR002100), likewise displaying more substantial increases of gene expression in hybrid seeds with *S. chilense* as maternal parent (supplementary table S6, supplementary fig. S2*E*, Supplementary Material online).

### Joint Signatures of Gene and sRNA Expression Dynamics

**Fig. 2.**
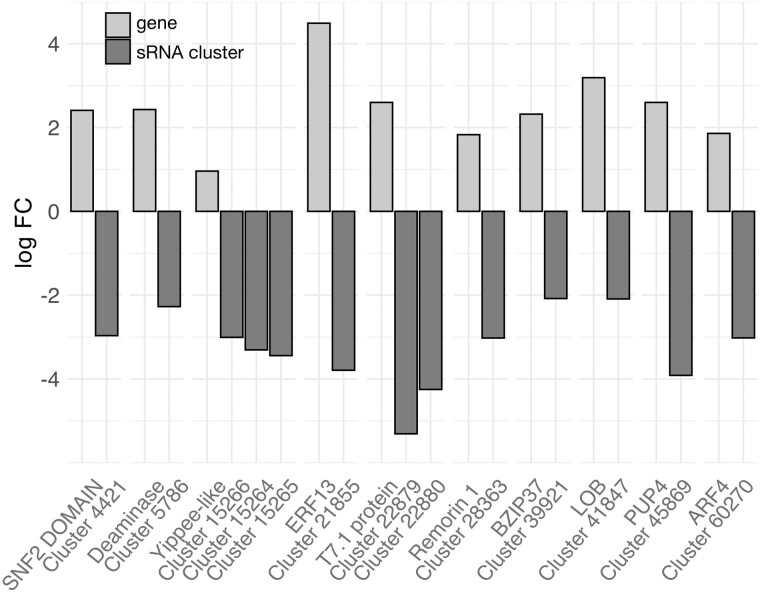
Lower seed transcript abundance of sRNA clusters is concomitant with significant upregulation of gene expression in hybrid endosperm. Negative fold changes of physically associated sRNA clusters are shown next to the positive fold changes of genes. From left to right, the genes shown are SNF2_N DOMAIN-CONTAINING PROTEIN (Solyc01g068320, PTHR45821: SF14), CMP/DCMP-TYPE DEAMINASE DOMAIN-CONTAINING PROTEIN (Solyc01g097880, PTHR11079: SF161), PROTEIN YIPPEE-LIKE (Solyc03g096150, PTHR13848: SF15), Ethylene-responsive transcription factor 13 (Solyc04g080910, ERF99_ARATH), T7.1 protein (Solyc05g012640, Q9FZE6_ARATH), REMORIN-LIKE (Solyc06g035920, PTHR31775: SF21), Transcription factor bZIP37 (Solyc08g074850, A4ZGR1_SOYBN), LOB domain protein family (Solyc09g014690, D7L292_ARALY), PURINE PERMEASE 4-RELATED (Solyc10g005160, PTHR31376: SF100), and Auxin response factor 4 (Solyc12g098460, D9HNT1_MAIZE). All changes in expression are significant with Bonferroni-corrected *P* values <0.05. log FC, log-fold-change.

To investigate the potential role of sRNAs in modulating gene expression in the endosperm, we integrated our seed sRNA data with our endosperm transcriptome data. sRNAs were given the annotation of the gene they mapped to if they fell within 2.5-kb boundaries (supplementary table S2, Supplementary Material online). Strikingly, the identity of many genes with mapped DE sRNAs revealed roles in epigenetic regulation and/or sRNA biogenesis, suggesting a regulatory feedback mechanism (supplementary tables S4 and S6, Supplementary Material online). We identified 30 DE sRNA clusters overlapping with 32 genes, in which underexpression of sRNAs in hybrid seeds was concomitant with significant overexpression of the corresponding genes in hybrid endosperms of both cross directions, PC and CP (fig. 2). Assembled information of gene and sRNA cluster expression and differential gene expression (DGE) is provided in supplementary table S4, Supplementary Material online. These particular cases suggest gene silencing by the reported clusters of sRNAs that appear to be partly defective in hybrid seeds. The tomato homolog of *DEFECTIVE IN MERISTEM SILENCING3* (*DMS3*) is *Solyc03g083120*. *DMS3* is a component of the canonical RdDM pathway in *Arabidopsis* (Matzke and Mosher 2014); we found it targeted by DE sRNA clusters in both species. Another member of the RdDM pathway targeted by DE sRNA clusters is *Solyc03g098280* (*SlAGO1b*), an *ARGONAUTE 1b* gene. ARGONAUTE proteins are core components of the sRNA-dependent silencing pathways (Matzke and Mosher 2014; Pikaard and Mittelsten Scheid 2014). Three DE sRNA clusters map within the boundaries of this gene; they are less expressed in hybrid seeds, concomitant with higher gene expression in the PC hybrid but slightly decreased gene expression in the CP hybrid.

Another putative member of the RdDM pathway with downregulated DE sRNAs and significant gene overexpression is *Solyc01g068320*, which encodes an SNF2 domain-containing protein related to CLASSY proteins (fig. 2, supplementary table S4, Supplementary Material online). Members of the CLASSY family have putative roles in RdDM (Law et al. 2011) and have recently been shown to be important regulators of sRNA production in *Arabidopsis* (Zhou et al. 2018). Other genes exhibiting clear signatures of TGS disturbance via RdDM upon hybridization are depicted in figure 2. Among these are *Solyc10g005160*, *PURINE PERMEASE 4* (*PUP4*), and two clustered *LATERAL ORGAN BOUNDARIES* (*LOB*) genes (*Solyc09g014700* and *Solyc09g014690*). Two genes (*Solyc02g091030*, *Solyc05g012640*) encode proteins with RNA and DNA polymerase activity, respectively, and are highly expressed in normal tomato endosperm, as is a gene encoding the *AUXIN RESPONSE FACTOR 4* (*ARF4*; *Solyc12g098460*), an important regulator of seed development.

**Fig. 3.**
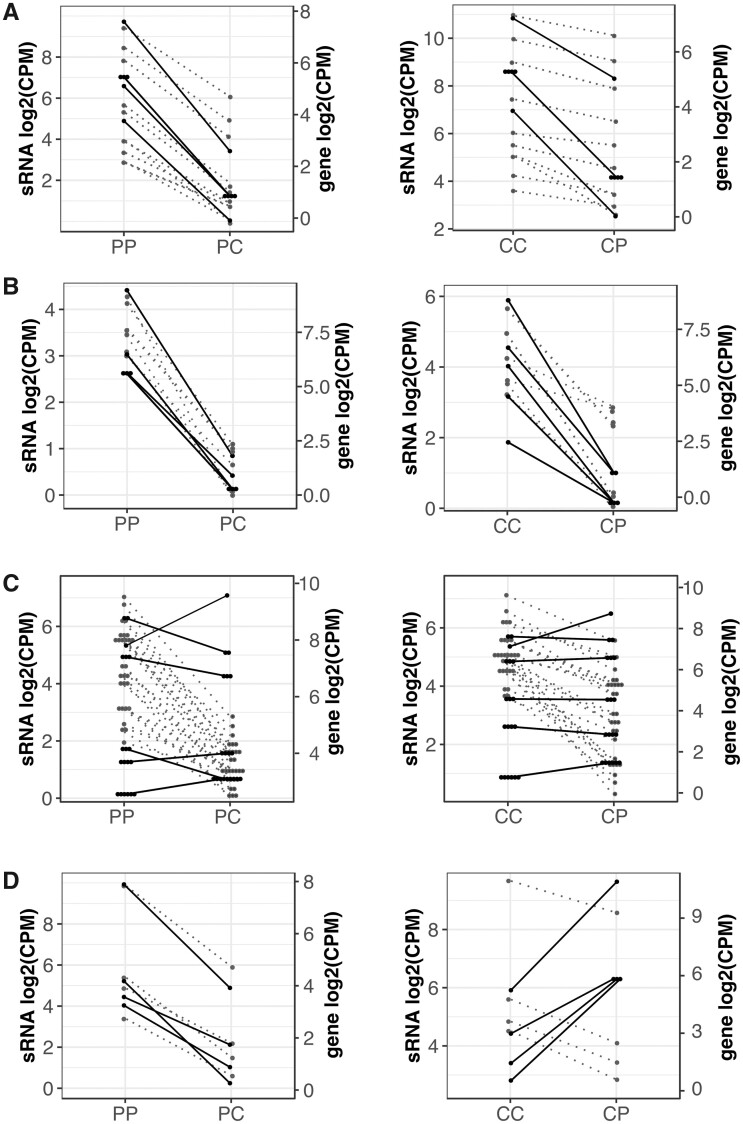
sRNA clusters that are DE upon hybridization target genes arranged in physical clusters. (A) Cluster on chromosome 5, *Lysine-specific histone demethylase 1 homolog 3* (*LDL3*) genes. (B) Cluster on chromosome 2 of genes coding for proteins belonging to the NUCLEAR TRANSPORT FACTOR 2 (PTHR10693: SF75) protein family. (C) Cluster on chromosome 3 of genes coding for proteins belonging to the YIPPEE domain (PTHR13848: SF5) protein family. (D) Cluster on chromosome 12 of genes belonging to the ARID DOMAIN-CONTAINING PROTEIN (PTHR15348: SF22) protein family. Dot plots show patterns of expression; on the left y axis sRNA expression and on the right y axis gene expression. Left and right panels show normal and hybrid seeds with *S. peruvianum* (P) and *S. chilense* (C) as maternal plants, respectively. Each dot represents an sRNA cluster and lines within single dot plots trace changes in expression between normally developing and hybrid seeds. Dotted lines and solid lines trace sRNA and gene expression, respectively. All plotted sRNA clusters are significantly DE. CPM, counts per million.

A striking result is that DE sRNAs map to genes arranged in clusters across the tomato genome (fig. 3, supplementary tables S4 and S7, Supplementary Material online); this leads to an increased number of genes per gene class consistently targeted by sRNAs. Therefore, the identity of genes located in physical clusters drives our enrichment analyses (supplementary table S6, Supplementary Material online). The most representative gene families have at least 11 and up to 28 genes with DE sRNA clusters mapping to them, these are HELICASE ATP-BINDING DOMAIN-CONTAINING PROTEIN (PTHR45821: SF15), PROTEIN YIPPEE-LIKE (PTHR13848: SF15), METHYL-CPG BINDING DOMAIN PROTEIN-LIKE, ISOFORM C (PTHR12396: SF0), NUCLEAR TRANSPORT FACTOR 2 (PTHR10693: SF75), and HELICASE C-TERMINAL DOMAIN-CONTAINING PROTEIN (PTHR45821: SF20) (supplementary tables S4 and S6, Supplementary Material online). These physically linked gene families have undergone expansions in the *Solanum* lineage compared to *Arabidopsis* (supplementary table S7, Supplementary Material online), and probably arose through instances of gene duplication and neofunctionalization. An example of this pattern are genes belonging to the protein Panther subfamilies NUCLEAR TRANSPORT FACTOR 2 (PTHR31413: SF10 and PTHR10693: SF75) with a single member in *Arabidopsis* (Mi et al. 2017). Nine of these genes are arranged in clusters on chromosome 2, with four having DE sRNAs mapping to them and a single Ninja-family protein targeted exclusively by 21–22-nt DE sRNAs (supplementary tables S4 and S7, Supplementary Material online). Eleven *LYSINE-SPECIFIC HISTONE DEMETHYLASE 1 HOMOLOG 3* (*LDL3*) genes have DE sRNAs mapped to them and have likewise expanded in *Solanum*, with seven members in contrast to the single one in *Arabidopsis* (Mi et al. 2017; supplementary tables S3, S4, and S7, Supplementary Material online).

Other genes arranged in clusters with sRNAs mapping to them are the chromatin remodeling protein families ARID DOMAIN-CONTAINING PROTEIN (PTHR15348: SF22) (Baba et al. 2011; Chandler et al. 2013), hereafter called ARID5 family, and HELICASE C-TERMINAL DOMAIN-CONTAINING PROTEIN (PTHR45821: SF20), including members of the aforementioned CLASSY protein family (supplementary table S4, Supplementary Material online). Other members of gene families occurring in clusters and targeted by DE sRNAs include genes encoding members of the Kinase protein family (D7MB90_ARALY) clustered on chromosome 12 and genes coding for proteins with a YIPPEE domain (PTHR13848: SF5) clustered on chromosome 3. The latter class of genes has been shown to play a role in the epigenetic regulation of chromatin, with conditional knockout mouse lines resulting in hypomethylated DNA and embryonic lethality (Kim et al. 2012; Subramanian et al. 2016).

Some of the genes targeted by DE sRNAs did not exhibit any detectable expression. Lack of expression may indicate that these sRNAs inhibit transcription of these genes, possibly via RdDM or related mechanisms leading to TGS or PTGS (Matzke and Mosher 2014; Pikaard and Mittelsten Scheid 2014; Cuerda-Gil and Slotkin 2016). For example, seven out of ten genes encoding a YIPPEE domain targeted by DE sRNAs were expressed in the endosperm. Among a large cluster of *DICERLIKE* genes on chromosome 1, we identified two (*Solyc01g009140*, *Solyc01g014450*) with DE sRNAs mapped to them; however, we did not detect any gene expression in the endosperm.

### Quantification of Expression Modes in Hybrid Endosperm and Seeds

**Fig. 4.**
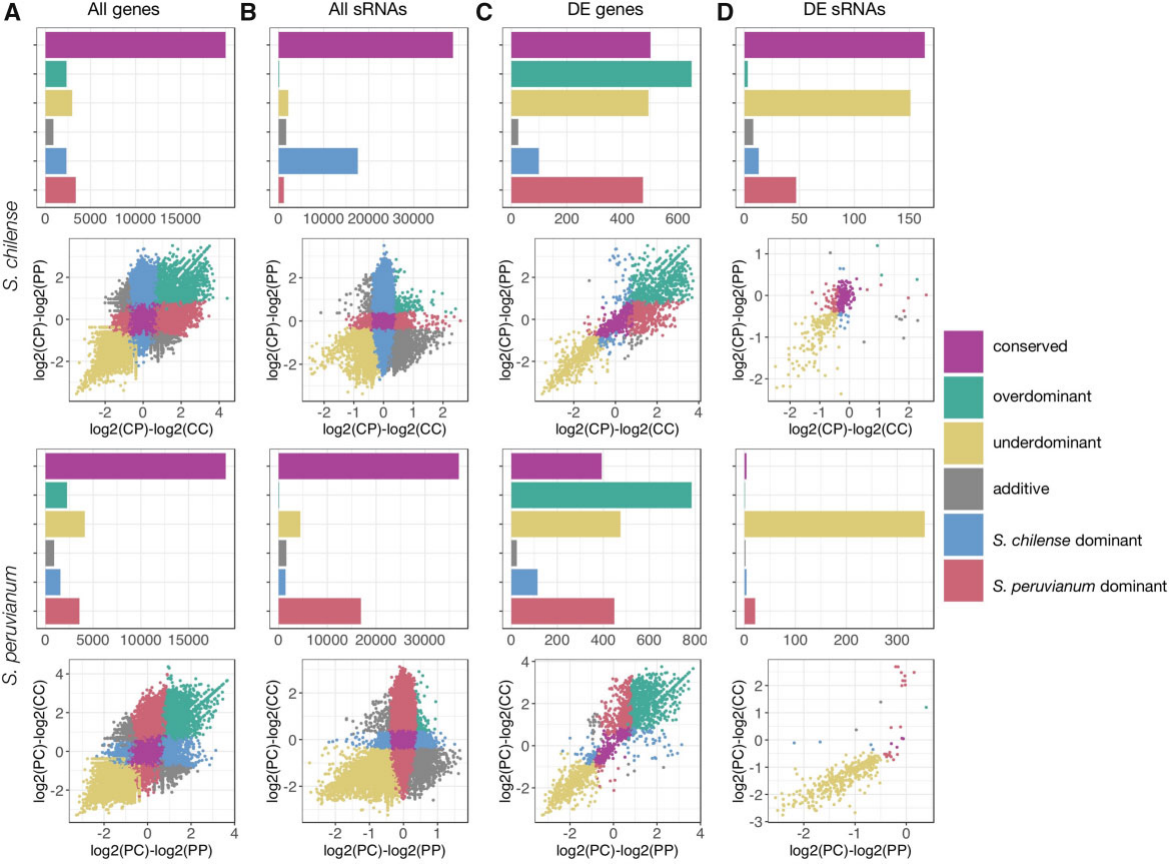
General patterns of expression modes. Hybrid versus normal seed comparisons with *S. chilense* (upper panel) and *S. peruvianum* (lower panel) as maternal parents, respectively. (A) All genes (*n *=* *33,805). (B) All sRNA clusters (*n *=* *31,189). (C) Differentially expressed genes (*n *=* *2,295). (D) Differentially expressed sRNA clusters (*n *=* *387). Expression mode categories are colored as follows: conserved, purple; overdominant, green; underdominant, yellow; additive, gray; *S. chilense* dominant, blue; *S. peruvianum* dominant, red.

We assessed the mode of expression (conserved, additive, dominant, overdominant, or underdominant) of sRNAs and gene transcripts by comparing total expression levels in *S. peruvianum*, *S. chilense*, and their reciprocal hybrids. Following the rationale described in previous studies (McManus et al. 2010; Combes et al. 2015), we performed analyses of expression modes for the DE transcripts and sRNAs as well as for the whole set of transcripts and sRNA clusters. The analysis of expression modes of all expressed genes and sRNA clusters (fig. 4*A* and *B*) revealed that a large proportion of these show conservation of parental (within-species) expression levels in the hybrids, particularly for gene expression (>60%; fig. 4*A*, purple). While conserved sRNA expression is also the dominant expression mode when evaluating all sRNAs (63% in *S. chilense* and 60.1% in *S. peruvianum*; fig. 4*B*), the entire sRNA data set also revealed a marked pattern of nonconservedness, with maternal dominance being a major category (28.6% in *S. chilense* and 27.4% in *S. peruvianum*; fig. 4*B*).

In both species, many DE genes show transgressive expression (fig. 4*C*), with overdominance being the predominant trend followed by underdominance of gene expression. Maternal dominance also markedly contributes to gene expression in the hybrids. An interesting result is the high proportion of genes that are in the *S. peruvianum*-dominant category in CP hybrid seeds, surpassing the maternal-dominant category for *S. chilense* (20.6% vs 4.3%; fig. 4*C*, upper panel). This result suggests that *S. peruvianum* in the paternal role greatly influences gene expression in CP hybrid endosperm despite contributing only one haploid genome. The signature of *S. peruvianum* dominance of gene expression in the CP hybrid is also evident in the expression mode of all genes and not only the DE genes (fig. 4*A*, upper panel). Although most genes show a conserved pattern of expression in both cross directions, the *S. peruvianum*-dominant category ranks second, surpassing other expression modes (10.5% *S. peruvianum* dominance; fig. 4*A*, upper panel). These results indicate that the “genomic dominance” of *S. peruvianum* relative to *S. chilense* is not restricted to DE genes but acts at a genome-wide level.

DE sRNAs are almost completely underdominant in PC hybrid seeds (fig. 4*D*). In contrast, many DE sRNA clusters show conserved expression in CP hybrid seeds. Although 133 sRNA clusters are consistently underdominant in both species, only 18 are exclusively underdominant in *S. chilense* in comparison to the 221 exclusively underdominant in *S. peruvianum*. The latter sRNA clusters have a mostly conserved mode of inheritance in *S. chilense*, with only 18% being *S. peruvianum*-dominant. The *S. peruvianum*-dominant signature evident in the mode of gene expression (fig. 4*A* and *C*) is also apparent in the expression mode of DE sRNA clusters in CP hybrid seeds, with 12.2% of the total set of DE sRNA clusters falling into this category. However, the most striking trend in the expression mode of sRNAs is that of underdominance of DE sRNA clusters in hybrid seeds (fig. 4*D*).

## Discussion

### Evidence for Conserved Epigenetic Landscapes in Compromised Hybrid Endosperm

Our analyses of sRNAs and transcripts that are DE between normal and failing seeds/endosperms revealed striking similarities with previous work on transcriptomic responses to hybridization in other taxa, particularly with the effects of Pol IV mutations on the epigenomic landscape of *Arabidopsis* endosperm. Erdmann et al. (2017) demonstrated that the Pol IV sRNA pathway mediates dosage interactions between maternal and paternal genomes. Specifically, they showed that disabling mutations in *nrpd1* induce shifts toward higher expression proportions of maternally inherited alleles. These results mirror our previous findings of increased maternal expression proportions in *Solanum* hybrid endosperms (Florez-Rueda, Paris, et al. 2016). Likewise, Erdmann et al. (2017) reported increased gene expression in *nrpd1* mutant endosperm compared to wild-type endosperm, resembling the increased gene expression among DE genes in *Solanum* hybrid endosperms (fig. 1*A*).

Taken together, the reduction in RNA Pol IV expression and the overall increase in expression of DE transcripts and maternal expression proportions in hybrids (Florez-Rueda, Paris, et al. 2016) allows us to draw comparisons between the effects of the *Arabidopsis nrpd1* mutant (Erdmann et al. 2017) and the natural case of HSF we explore in *Solanum*. Based on these obvious parallels, we postulate a *Solanum* Pol IV sRNA pathway acting in a similar fashion to that described in *Arabidopsis* (Erdmann et al. 2017; Satyaki and Gehring 2019), mediating dosage interactions of the parental genomes upon fertilization. We propose that the Pol IV sRNA pathway serves to maintain the 2m:1p ratio expected from the endosperm’s genomic constitution, likely through direct and/or indirect effects on many genes in the endosperm. The observed reduced expression of the main Pol IV subunits may be functionally linked to the increased maternal expression proportions in the hybrid endosperm of wild tomatoes (Florez-Rueda, Paris, et al. 2016).

Increased expression of MADS-box TF genes upon hybridization has previously been reported in *Arabidopsis* (Josefsson et al. 2006; Walia et al. 2009; Hehenberger et al. 2012; Lu et al. 2012; Burkart-Waco et al. 2013), *Capsella* (Rebernig et al. 2015), and *Oryza* (Ishikawa et al. 2011). We found a large number of MADS-box genes (among other TF genes) overexpressed in both reciprocal hybrid endosperms (supplementary fig. S2*E*, Supplementary Material online; supplementary table S3, Supplementary Material online). MADS-domain TFs have been shown to play key regulatory roles in plant reproduction, in particular in regulating female gametophyte, embryo, and endosperm development (reviewed in Masiero et al. 2011). Likewise, the *AGAMOUS-LIKE* (*AGL*) MADS-box TF genes were jointly overexpressed in “paternal-excess-like” crosses involving *Solanum chilense*, *S. peruvianum*, and *S. arcanum* (Roth et al. 2019). These TFs are part of the GO protein dimerization activity (GO: 0046983) and include 11 *AGL* genes, 13 *2FE-2S FERREDOXIN-LIKE* genes, three *PHERES* genes, *APETALLA3*, and *SEPALATA3*, among others (supplementary tables S3 and S6, Supplementary Material online). AGL proteins have been shown to affect endosperm development in *Arabidopsis* (Kang et al. 2008; Shirzadi et al. 2011). Intriguingly, overexpression of *AGL62* and *AGL90* is associated with the postzygotic barrier between *A. thaliana* and *A. arenosa*, which manifests itself as endosperm over-proliferation and delayed cellularization (Josefsson et al. 2006; Walia et al. 2009; Burkart-Waco et al. 2013). Transgenic underexpression of *AGL62* attenuates the level of HSF in *Arabidopsis* (Hehenberger et al. 2012), thus providing functional validation for this pattern.

sRNAs have been shown to modulate the expression of MADS-box TF genes; maternal sRNA expression is negatively correlated with *AGL* gene expression in *Arabidopsis* endosperm (Lu et al. 2012). However, our analyses do not support a consistent trend of sRNAs targeting MADS-box TF genes; we did find three MADS-box genes with associated DE sRNA clusters (*Solyc03g062820.1*, *Solyc10g012180.1*, and *Solyc10g018110.1*) (supplementary table S4, Supplementary Material online). Taken together, this and earlier *Arabidopsis* studies suggest that the putative functions of MADS-domain TFs in mediating both normal seed development and endosperm-based HSF are conserved across angiosperms. Specific functions of MADS-box TF genes in *Solanum* have not yet been studied, but here we have uncovered a list of candidate genes with potentially important roles that remain to be functionally validated.

Qualitative and quantitative sRNA differences between the parental genomes may affect the hybrid expression of genes and TEs neighboring the sRNAs. However, our analyses did not uncover significant associations between DE sRNAs and nearby TEs (data not shown). In some instances of hybridization, changes in sRNA expression are concomitant with heterosis (Groszmann et al. 2011; Barber et al. 2012), although a causal role of sRNAs has not been shown; in *Solanum* and other plant genera, such expression changes may lead to HSF (Ng et al. 2012; Florez-Rueda, Paris, et al. 2016; Garner et al. 2016). We found that DE sRNAs were consistently underexpressed in hybrid seeds (fig. 1*B*); this trend is reflected in underdominance of sRNA expression in hybrid seeds when compared to seeds derived from intraspecific crosses on the same maternal plant. Underdominance of sRNA expression upon hybridization has been reported in other tissues besides the seed in diverse plant genera (Groszmann et al. 2011; Barber et al. 2012; Lu et al. 2012; Shen et al. 2012; Shivaprasad et al. 2012; He et al. 2013). In all these examples as well as ours, the molecular mechanisms leading to reduced sRNA levels are unknown; based on the reduced expression of Pol IV subunits (supplementary table S4, Supplementary Material online), we hypothesize that perturbations in the Pol IV sRNA pathway may be involved (Erdmann et al. 2017; Satyaki and Gehring 2019).

We uncovered high levels of maternal dominance of sRNA expression that may be explained by the nature of the seed tissue we collected (manually extracted seeds with subsequent washes), the maternal seed coat being one of its components; recent data in *Brassica rapa* indicate high expression of a small subset of 24-nt sRNAs in ovule and seed coat tissues (Grover et al. 2020). Another possible scenario is that the sRNAs exhibiting maternal dominance may be generated by filial seed tissues. However, there is disagreement among studies in *A. thaliana* and *B. rapa* whether 24-nt sRNAs show strongly maternally biased expression (Mosher et al. 2009; Erdmann et al. 2017; Satyaki and Gehring 2019; Grover et al. 2020). Regardless, these sRNAs are thought to accumulate in the endosperm and to mediate gene expression (Calarco and Martienssen 2011); the high level of observed maternal dominance in the expression inheritance of sRNAs in both species suggests that this may also be the case in *Solanum*.

### Feedback Regulation of Core Silencing Proteins through sRNA-Mediated Silencing

Our data suggest that sRNAs that are DE in hybrid seeds target many genes with important functions in sRNA biogenesis and epigenetic regulation. Importantly, we show members of the *ARID5* and *CLASSY3* families, *DICER*, *AGO1B*, and *DMS3* to be associated with sRNAs in tomato seeds in abundances that are significantly different in PC vs CP hybrid seeds (supplementary table S4, Supplementary Material online). For some of these genes, we were able to additionally assess gene expression levels; the apparent effect of sRNA abundance on gene expression suggests that sRNA-mediated gene silencing impacts the expression of some of these genes and may be defective in hybrid seeds, plausibly contributing to HSF. We hypothesize that these genes, some of which are regulators of TGS or PTGS themselves, are subject to feedback regulation orchestrated by their own sRNA products. Negative feedback regulation of *DICERLIKE* genes has been described in *Arabidopsis* (Xie et al. 2003; Borges and Martienssen 2015) and yeast (Oberti et al. 2015); such feedback regulation is thought to allow homeostatic control of the cellular silencing machinery (Borges and Martienssen 2015). The only gene for which we detected an effect on allele-specific expression is the ARGONAUTE-encoding gene *Solyc03g098280*, *SlAGO1b*. As a paternally expressed gene (PEG) with low maternal proportions in the normal endosperm of *S. peruvianum*, it showed the “typical” increase (from 0.25 to 0.87 maternal proportion) that we previously uncovered for the majority of PEGs in the “maternal-excess-like” PC hybrid endosperm (Florez-Rueda, Paris, et al. 2016). We posit that the observed underexpression of sRNA clusters mapping to *SlAGO1b* and its flanking regions may be responsible for its increased gene expression, with a higher maternal proportion in hybrid endosperm derived from maternal *S. peruvianum*.

Although we cannot provide functional verifications to support feedback regulation of genes involved in sRNA-mediated gene silencing in *Solanum* endosperm, our results provide pioneering glimpses into the epigenetic landscape in the context of HSF. We show that DE sRNA clusters map to genes playing pivotal roles in epigenetic regulation, with expected implications for HSF. Further characterization of the epigenomic landscape of the endosperm through chromatin immunoprecipitation and sequencing (ChIP-seq) as well as methylome sequencing will allow a proper evaluation of these hypotheses.

### Mode of Expression in Hybrids: Dominance May Reflect Differences in Effective Ploidy

Previous analyses of expression modes have been restricted to evaluating inheritance in whole plants that were successful hybridization products of within- or among-species crosses (Eichten et al. 2011; Shi et al. 2012; Bell et al. 2013; Combes et al. 2015; Li et al. 2015; Carlson et al. 2017). Although these types of analyses on whole hybrid plants provide valuable insights into the transcriptomic effects of hybridization, they do not address the issue of parental conflict that is expected to play out in the developing seed (Haig and Westoby 1991; Haig 2013; Lafon-Placette and Köhler 2016; Städler et al. 2021).

The near-complete HSF phenotype characterizing both cross directions between *S. peruvianum* and *S. chilense* (yet with marked phenotypic differences between reciprocal crosses) may be seen as resulting from different levels of parental conflict within each of the parental lineages (Brandvain and Haig 2005; Haig 2013; Städler et al. 2021). Hybrid seeds from *S. chilense* maternal plants (CP) are larger, showing a “paternal excess-like” phenotype in contrast to the smaller hybrid seeds with *S. peruvianum* mothers (PC) that show a “maternal excess-like” phenotype (Florez-Rueda 2014; Florez-Rueda, Paris, et al. 2016; Roth, Florez-Rueda, Griesser, et al. 2018). The reciprocal differences both in early seed development and mature hybrid seed size suggest that the *S. peruvianum* lineage evolved under higher levels of parental conflict than has *S. chilense.* These patterns and inferences are consistent with higher range-wide nucleotide diversity, indicative of higher effective population size (Städler et al. 2008; Tellier et al. 2011; Beddows et al. 2017), and higher expression levels of imprinted genes in *S. peruvianum* (Roth, Florez-Rueda, Paris, et al. 2018). Similar conclusions have been reached in studies of compromised hybrid endosperm and seed development in the *Mimulus guttatus* complex (Coughlan et al. 2020).

Likewise, *S. peruvianum* drives expression landscape polarization in hybrid endosperms derived from reciprocal crosses with both *S. chilense* and *S. arcanum* (Roth et al. 2019). In line with these observations, our analyses of the expression modes of DE sRNAs and genes revealed a trend of *S. peruvianum* dominance in CP hybrid seeds and endosperm, respectively (fig. 4*C* and *D*, upper panel). This signature holds true not only for DE genes and sRNAs but also at a genome wide-level, specifically in the larger data set of all expressed genes where the *S. peruvianum*-dominant category ranks second (fig. 4*A*, upper panel). We interpret the pattern of *S. peruvianum* dominance as consistent with the rationale of the weak inbreeder/strong outbreeder (WISO) hypothesis (Brandvain and Haig 2005), with the *S. peruvianum* genome “overpowering” that of *S. chilense*, which putatively evolved under lower levels of parental conflict. These inferences are in accordance with our prior and current evidence for higher effective ploidy of *S. peruvianum* compared to *S. chilense* (Roth et al. 2019; Städler et al. 2021), and how it plausibly underpins the developmental and phenotypic differences of seeds between these two wild tomato lineages.

## Materials and Methods

### Plant Material, RNA Extraction, and Library Preparation

All seeds were obtained from the C.M. Rick Tomato Genetics Resource Center at U.C. Davis (http://tgrc.ucdavis.edu, last accessed June 16, 2016). For *S. peruvianum*, we used seeds from accession LA1616 (Dept. Lima, Peru) and for *S. chilense*, we used seeds from accession LA4329 (Region Antofagasta, Chile). We used four individual plants, referred to as 1616A, 1616J, 4329B, and 4329K and analyzed three different parental combinations: the within-species *S. peruvianum* case (PP) with plants 1616A and 1616J as parents, the within-species *S. chilense* case (CC) with plants 4329B and 4329K as parents, and the hybrid cases (PC and CP) with plants 1616A and 4329B in both parental roles in reciprocal crosses. The parental plants were grown from seeds and transferred to a climate chamber before the onset of the experiments. The conditions in the climate chamber were 12 h light (18 klux) at 22°C with 50% relative humidity and 12 h darkness (0 klux) at 18°C with 60% relative humidity. For each of the three cross types, hand pollinations were performed and developing fruits were collected on each plant for each cross type.

Based on prior studies of seed development in *Solanum* (e.g., Beamish 1955; Briggs 1993) and our own histological analyses (Roth, Florez-Rueda, Griesser, et al. 2018), we chose an early globular embryo stage to collect the material for library preparation. We thus collected fruits 14 days after pollination (DAP), always in the late afternoon. This developmental stage was chosen because it was early enough to distinguish the developing embryo from the surrounding endosperm tissue, while the latter was large enough to extract RNA in the quantities needed for library preparation. For each plant and cross type, two separate mRNA libraries were prepared from endosperm tissue, for a total of 12 endosperm libraries. The raw data for the endosperm transcriptomes have been published; detailed methodology for its production is described in Florez-Rueda, Paris, et al. (2016). In brief, fruits were harvested, fixed, and endosperms were laser captured with the LAM technique outlined in Florez-Rueda, Grossniklaus, et al. (2016).

The same crossing design described above for endosperm transcriptomes was implemented for the whole-seed sRNA data set. As we were interested in overall—rather than parent-specific—sRNA expression levels and sRNAs were found to be abundant in all three *Arabidopsis* seed compartments (Erdmann et al. 2017; Kirkbride et al. 2019; Satyaki and Gehring 2019), we extracted sRNAs from whole seeds. Moreover, we generated sRNA libraries only from hybrid and normal seeds from plants 1616A and 4329B, that is, those serving as parents in both intra- and interspecific crosses (supplementary fig. S1, Supplementary Material online). For these sRNA libraries, we generated three replicates for our analyses, each replicate reflecting independent hand-pollination events performed on different days. As for the endosperm transcriptomes, developing fruits were collected at 14 DAP in the late afternoon and immediately placed into RNA later solution. The samples were immediately transferred to a refrigerator and remained in the RNAlater solution for a minimum of 24 h and a maximum of 48 h. Whole seeds were dissected in RNase-free water and subjected to consecutive water washes to remove the fruit flesh debris. We collected a minimum of 1 mg of seeds from tens of fruits from each cross type and proceeded to sRNA extraction. RNA was extracted using the miRVana RNA isolation kit (Ambion, Life Technologies Corporation, Foster City, CA, USA). sRNA libraries were prepared using the NEXTflex SRNAs-Seq Kit v2 according to the manufacturer’s protocol (Bioo Scientific Corporation, Austin, TX, USA). Libraries were sequenced in single-end fashion on one lane of an Illumina HiSeq 4000 at the Functional Genomics Center Zurich (www.fgcz.ch).

### Read Mapping and Differential Expression Analyses

Mapping of sRNA reads was performed using ShortStack (Axtell 2013), using default options (–mincov 0.5 rpm –pad 75) and allowing no mismatches to the SL2.50 assembly of the cultivated tomato reference genome (The Tomato Genome Consortium 2012) deposited in ensemble genomes (https://plants.ensembl.org/Solanum_lycopersicum/Info/Annotation/#genebuild, last accessed February 7, 2017). We additionally mapped our sRNA reads with two mismatches allowed, under the rationale that our target species (which share the same divergence time from the cultivated tomato) may exhibit slight sequence divergence from each other and/or from the cultivated tomato. However, given that the results qualitatively agree (data not shown), we have opted to base all results presented in this article on the more conservative option of zero mismatches allowed. To minimize possible biases due to multimapping reads, we performed analyses using ShortStack v3’s “Unique” weighting option (–mmap u), which has been shown to outperform alternative mapping options (Johnson et al. 2016). Briefly, multimapping reads are assigned their mapping positions in a probabilistic manner, taking into account the local density of uniquely aligned sequence reads (Johnson et al. 2016). Subsequently, sRNA clusters were delimited according to ShortStack’s cluster definition method which entails a two-step process. First, local “islands” of significant alignment coverage are identified (based on the –mincov option), which in a second step may be joined with adjacent islands to form clusters (based on the –pad option) (Axtell 2013). We delimited these sRNA clusters using BEDTools window command (Quinlan and Hall 2010) and used them for further analyses. Based on the corresponding SL2.50 ensemble annotation of the reference genome, we classified 1,619 sRNAs as miRNAs; ShortStack inferred 54 miRNAs for a total of 1,646 miRNAs. Other forms of noncoding RNAs represented in our sRNA libraries include 1,349 antisense RNAs, rRNAs, tRNAs, snoRNAs, snRNAs, and SRPRNAs. These latter forms were removed before performing differential expression analyses. By using the counts obtained by ShortStack, we performed DGE analyses using DESeq2 (Love et al. 2014) in the same manner as for the endosperm transcriptomes (see below).

We reanalyzed the endosperm transcriptome data previously produced (Florez-Rueda, Paris, et al. 2016). Raw reads were mapped to the SL2.50 assembly of the tomato genome deposited in ensemble genomes (https://plants.ensembl.org/Solanum_lycopersicum/Info/Annotation/#genebuild). The tuxedo pipeline (Trapnell et al. 2012) was used for the assembly of reads, mapping to the tomato reference genome, and count estimation. Raw count tables were produced with additional packages of the Tuxedo pipeline, cuffquant and cuffnorm; unnormalized counts per transcript were used for subsequent analyses. DGE analyses for transcripts as well as for sRNA clusters were performed using DESeq2 (Love et al. 2014), as implemented in the RNAseqWrapper package (Schmid 2017) in R (R Development Core Team 2014). To test for DGE between viable and hybrid seeds while taking into account expression variation within both species, a model of a single factor with multiple levels (species correspondence: *S. peruvianum*, *S. chilense*, and type of seed: normal, hybrid) was implemented in the given RNAseqWrapper module (Schmid 2017). This implies that we contrasted all within-species expression data as one entity (from crosses PP and CC) with all hybrid expression data as the other entity (from crosses PC and CP). DE transcripts and sRNAs with more than absolute 2.5 and 2 log fold-change, respectively, and a Bonferroni-corrected *P* value <0.05 are reported as significantly DE. For the sets of DE genes and sRNAs between the combined within-species versus hybrid data, we compared expression levels via a Wilcoxon rank-sum test.

Downstream gene enrichment analyses were carried out using the STRING database (Szklarczyk et al. 2017). We report functional enrichment analyses from STRING with a FDR of 0.01. When reported, GO assignment was assessed using the PANTHER database (Mi et al. 2017). These two databases, STRING and PANTHER, were used for fine-tuning annotation of genes lacking annotation in the corresponding SL2.50 ensemble functional annotation files. We refer to gene clusters when three or more genes with the same annotation are located within 5 kb of genomic space. This delimitation is based on shared features of their curated joint annotation; nevertheless, genes within a gene cluster may differ in structural annotation and are not necessarily identical copies of the same gene.

### Expression Mode Classification

We compared expression levels of gene transcripts and sRNA clusters among *S. peruvianum* (PP), *S. chilense* (CC), and their reciprocal hybrids (PC and CP), following the rationale of previous studies to discriminate among the various categories of expression modes (McManus et al. 2010; Combes et al. 2015). Irrespective of whether a gene or sRNA was found to be DE, genes with less than 1-fold change between normal and hybrid endosperm were considered to exhibit *conserved* expression; for sRNAs, we used a lower threshold of 0.5-fold expression change. The mode of expression was inferred to be *additive* if expression level in the hybrids was less than in *S. peruvianum* but greater than in *S. chilense* (or vice versa). If hybrid expression was similar to one of the parental species it was classified as *dominant* for the respective species, and genes and sRNAs with either higher or lower hybrid expression than in both *S. peruvianum* and *S. chilense* were classified as exhibiting *overdominant* and *underdominant* expression, respectively.

## Data Availability

Raw sequence data for the RNA-sequencing data set used in this study are available from the Sequence Read Archive (https://trace.ncbi.nlm.nih.gov/Traces/sra/) with the accession numbers PRJNA713528 (sRNAs; this study) and SRX1850236 (mRNA; Florez-Rueda, Paris, et al. 2016).

## Supplementary Material

Supplementary data are available at *Genome Biology and Evolution* online.

## Supplementary Material

evab107_Supplementary_Data
